# The association between bilirubin levels, and the incidence of metabolic syndrome and diabetes mellitus: a systematic review and meta-analysis of cohort studies

**DOI:** 10.1186/s40842-023-00159-0

**Published:** 2024-01-10

**Authors:** Maziar Nikouei, Mojtaba Cheraghi, Faezeh Ghaempanah, Parisa Kohneposhi, Nadia Saniee, Sirous Hemmatpour, Yousef Moradi

**Affiliations:** 1 Kurdistan University of Medical Sciences, Sanandaj, Iran; 2 Department of Public Health, Asadabad School of Medical Sciences, Asadabad, Iran; 3 Department of Pediatrics, School of Medicine, Besat Hospital, Kurdistan University of Medical Sciences, Sanandaj, Iran; 4 Social Determinants of Health Research Center, Research Institute for Health Development, Kurdistan University of Medical Sciences, Sanandaj, Iran; 5 Department of Epidemiology and Biostatistics, Faculty of Medicine, Kurdistan University of Medical Sciences, Sanandaj, Iran

**Keywords:** Bilirubin level, Diabetes, Metabolic syndrome

## Abstract

**Objectives:**

The objective of this meta-analysis was to investigate the association between plasma bilirubin levels and the incidence of metabolic syndrome and diabetes mellitus across all populations.

**Methods:**

Several databases were searched, including PubMed (Medline), Scopus, Web of Science, and Embase (Elsevier), to identify relevant cohort studies. All cohort studies that reported the risk ratio along with a 95% confidence interval were included. The association between bilirubin levels and metabolic syndrome or diabetes was reported as a pooled RR with a 95% CI in the forest plot. All analyses were conducted using STATA version 17, with a significance level of 0.05.

**Results:**

Out of the 10 studies included in the analysis, four investigated the effect of hyperbilirubinemia on the incidence of type 2 diabetes. When these four studies were combined, the pooled RR was 0.78 (95% CI: 0.73, 0.83; I^2^: 88.61%; *P*_heterogeneity_ <  0.001), indicating a significant association between hyperbilirubinemia and decreased risk of type 2 diabetes. Five of the 10 studies evaluated the effect of hyperbilirubinemia on the incidence of metabolic syndrome, and the pooled RR was 0.70 (95% CI: 0.67, 0.73; I^2^: 78.13%; *P*_heterogeneity_ <  0.001), indicating a significant association between hyperbilirubinemia and decreased risk of metabolic syndrome.

**Conclusion:**

The findings suggest that elevated levels of bilirubin may have a significant protective effect against the development of diabetes mellitus and metabolic syndrome.

## Introduction

Metabolic syndrome (MetS) is a cluster of clinical symptoms that often occur together, although this co-occurrence is not necessarily due to chance in a patient [1, 2]. In 1998, the World Health Organization (WHO) introduced criteria to establish a unified concept of MetS and provide a practical tool for clinicians and researchers. These criteria included diabetes, impaired fasting glucose, glucose intolerance, or insulin resistance, along with the presence of two or more other criteria, including a body mass index (BMI) greater than 30, waist circumference greater than 0.9 m for men and 0.85 m for women, serum triglycerides greater than 150 mg/dL, blood pressure greater than 90/140 mmHg, microalbuminuria greater than 20 μg/min, and low levels of high-density lipoprotein (less than 50 mg/dL) [2, 3].

The National Cholesterol Education Program’s Adult Treatment Panel III (NCEP: ATP III) proposed additional criteria to define MetS. According to this definition, a person is classified as having MetS if they have three or more of the specified criteria [3]. These criteria include central obesity (waist circumference greater than 102 cm for men and 88 cm for women), triglycerides greater than 1.7 mmol/l, HDL < 1 mmol/l for men and HDL < 1.3 mmol/l for women, blood pressure greater than or equal to 135.85 mmHg and fasting plasma glucose greater than or equal to 6.1 mmHg [3, 4].

Over the past two decades, there has been a sudden increase in the prevalence of MetS worldwide, and conflicting reports have been presented [5]. These contradictions may be attributed to differences in gender, age, race, socioeconomic status, and the definition used to identify MetS [1, 6]. Despite the differences in studies, it is estimated that approximately 1.3 to 1.4% of adults worldwide meet the criteria for MetS. Regardless of the specific criteria used, the prevalence of MetS is high and increasing globally. According to the NCEP: ATP III criteria, it is estimated that about 34% of people worldwide have MetS [1, 2, 7, 8]. Similar to Western societies, the prevalence of MetS is also sharply increasing in developing countries. This rate varies from 9.8% in men living in northern regions of India to 42% in Iranian women [5, 9, 10]. Studies conducted in European countries have estimated the prevalence of MetS to be 36% in men and 22% in women. Additionally, due to the increasing prevalence of diabetes and obesity worldwide, it can be inferred that the prevalence of MetS is also increasing globally [11]. The diagnosis of MetS in a person predicts the risk of other diseases, especially cardiovascular ones, stroke and diabetes [12]. Based on a large number of studies, patients with MetS have been found to have coronary artery disease (CAD) and its risk is higher in patients with MetS than other people in the society [13]. People with MetS are at a higher risk of myocardial infarction and heart attack, resulting in a higher death rate from CAD compared to those without the syndrome [12–14]. Non-diabetic individuals with MetS are also at a higher risk of developing type 2 diabetes, with a 5 times higher probability than those without the syndrome [12–14]. Identification of factors influencing the incidence of MetS in a society is crucial due to its association with important diseases, particularly type 2 diabetes. The prevalence of type 2 diabetes is rapidly increasing in the United States and worldwide, and early identification and prediction of this disease are essential due to its significant complications and costs. Diabetes is an important criterion for the diagnosis of MetS according to the definitions of WHO, ATPIII, and IDF, and it may also be considered one of the syndrome’s important complications [15, 16]. Preliminary studies have suggested that the level of bilirubin in the body may serve as an important predictor for diabetes and MetS [16]. Bilirubin is the final product of metabolism, and 80% of it in the body is derived from the breakdown of hemoglobin in red blood cells through the reticuloendothelial cycle. Due to its potent antioxidant properties, mild hyperbilirubinemia may play a protective role against ischemic heart disease and cancer [17]. Numerous studies have examined the link between plasma bilirubin levels and the incidence of MetS and diabetes. Some of these studies have reported a protective association between high plasma bilirubin levels and the incidence of MetS and diabetes mellitus (DM) [18]. However, other studies have reported the opposite or did not show a significant association between bilirubin levels, MetS, and DM [18–20]. The conflicting data regarding the association between bilirubin levels and the incidence of MetS or DM has made it challenging to develop appropriate treatment guidelines for this issue. To develop accurate guidelines based on valid evidence, systematic reviews and meta-analyses are necessary to combine the published evidence from primary studies with conflicting data. Therefore, this meta-analysis was designed to investigate the association between plasma bilirubin levels and the incidence of MetS and DM.

## Methods

The current investigation represents a systematic review and meta-analysis that involved six fundamental stages, namely Search Syntax and Search Strategy, Screening, Selection, Data Extraction, Quality Assessment, and Meta-Analysis. This study was conducted in compliance with the Preferred Reporting Items for Systematic Reviews and Meta-Analyses (PRISMA) framework [21].

For the current meta-analysis, the primary keywords and their corresponding synonyms were identified through a comprehensive search of MeSH, Thesauruses, and EMTREE. The search was carried out across several databases, including PubMed (Medline), Scopus, Web of Science, and Embase (Elsevier). The search was limited to articles published between January 2015 and August 2023. The selected time period for this meta-analysis was based on a prior meta-analysis published in 2016, which covered articles published until the beginning of 2015. In an effort to provide updated findings, the present study was designed and conducted to extend the search period and include relevant studies published between January 2015 and August 2023 [22]. In order to conduct the research, keywords related to bilirubin including “Delta-Bilirubin”, “Calcium Bilirubinate”, “Bilirubin”, “Hematoidin”, “Monosodium Salt Bilirubin”, “Disodium Salt Bilirubin” were combined with keywords related to the desired outcome, such as “Diabetes Mellitus”, “Metabolic Syndrome”, “Insulin Resistance Syndrome”, “Metabolic Cardiovascular Syndrome”, “Type 2 Diabetes”, “Non-Insulin-Dependent Diabetes Mellitus”, “Adult-Onset Diabetes Mellitus”, and “Stable Diabetes Mellitus” to be searched in the desired databases.

Following the search, all results were imported into Endnote version 8. Duplicate studies were initially removed by assessing the title, authors, and year of publication of the articles. Subsequently, the screening stage was conducted, which involved evaluating the title, abstract, and full text of the remaining articles. Based on the predetermined inclusion and exclusion criteria, articles that were not relevant to the subject and purpose of the study were excluded from the review. In addition to searching international databases, a manual search was also performed to identify relevant articles. The references of selected studies were reviewed, and any similar studies found were included in the analysis.

### Inclusion and exclusion criteria

The study’s inclusion criteria were established using the PECOT structure. Specifically, case-control and cohort studies exploring the relationship between bilirubin levels, MetS, and types of diabetes were included. Cohort studies focusing on the entire population, encompassing individuals with both impaired and normal bilirubin levels, and investigating various bilirubin levels as the exposure, with MetS and types of diabetes as desired outcomes, were also incorporated into the meta-analysis. Conversely, other study types such as review studies, case reports, case-control studies, cross-sectional studies, clinical trials, other interventional studies, and letters to the editor were excluded from this research. In instances where the full text of a study meeting the inclusion criteria was unavailable, the authors were contacted via email to request the full text. Studies for which no response was received were excluded. The process of selecting and screening articles for this meta-analysis was independently conducted by two authors.

### Data extraction and qualitative assessment of articles

Upon completion of the screening stage using the inclusion and exclusion criteria, an information extraction checklist was developed to extract relevant information from the final articles. The checklist included publication years, studied populations, average age, sample sizes, types of bilirubin investigated, study results, and effect sizes. Two authors conducted a qualitative evaluation of the studies using the Newcastle-Ottawa Quality Assessment Scale (NOS) checklist, which is designed to assess the quality of observational studies. The NOS checklist consists of 8 items grouped into three categories, including the selection of study samples, the comparison and analysis of study groups, and the measurement and analysis of the desired outcome. Each item was given a score of one if observed in the study, and the maximum score for each study was 9 points. In the event of discrepancies in the assigned scores, a discussion method and a third researcher were utilized to reach a consensus.

### Statistical analysis

To assess the association using pooled risk ratio (RR) with a 95% confidence interval, the meta set command was utilized, taking into account the logarithm and standard deviation of the logarithm of the RR. Heterogeneity among studies was evaluated using the I2 value and Cochrane’s Q test. Cochrane’s criteria were applied, where 0 to 25% signified the absence of heterogeneity, 25 to 50% indicated low heterogeneity, 50 to 75% indicated high but acceptable heterogeneity, and 75 to 100% indicated high and unacceptable heterogeneity. Egger’s test and funnel plot were used to assess publication bias. Subgroup analyses were performed based on the reporting of important variables in selected cohort studies. Statistical analysis was performed using STATA 16.0, and a *P*-value < 0.05 was considered statistically significant.

## Results

### Search results

**Fig. 1:**
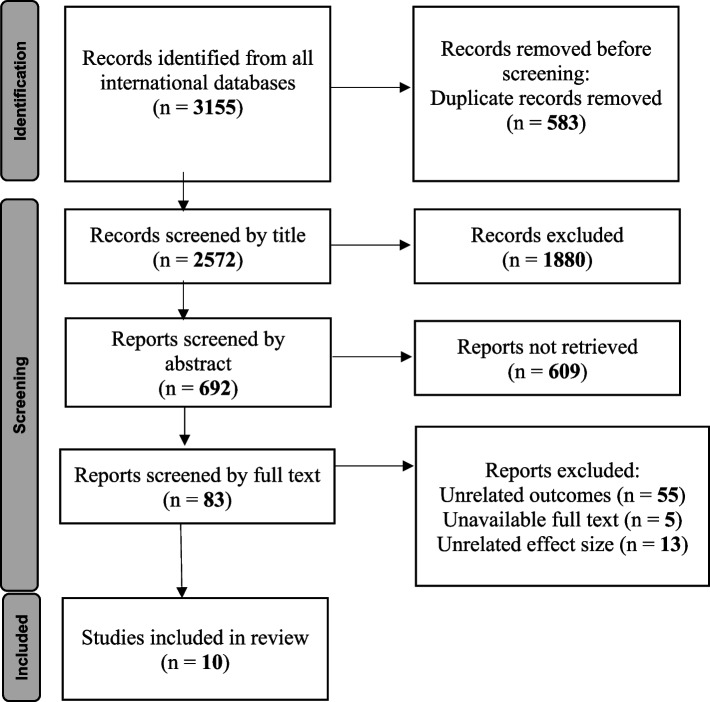
Flow diagram for related article numbers which included in meta-analysis

Following the search, a total of 3155 studies were identified, of which 583 were excluded due to duplication. The remaining 2572 studies underwent initial screening based on the title. After this stage, 692 studies were retained and underwent further screening based on the abstract. Following this step, 83 studies remained, which were then screened based on the full text. Finally, the meta-analysis was conducted based on the results of 10 cohort studies (Fig. 1).

### Qualitative results

The general characteristics of the 10 studies included in the meta-analysis are presented in Table 1. The total sample size of these studies was 79,508 individuals, and all were cohort studies that primarily utilized the TBIL (total bilirubin) index to measure bilirubin levels. The desired outcomes in these studies included the incidence of gestational diabetes (GDM), type 2 diabetes mellitus (T2DM), and MetS. Specifically, 4 studies with 34,638 participants investigated the effect of hyperbilirubinemia on type 2 diabetes, 5 studies with 43,735 participants examined the effect of hyperbilirubinemia on MetS, while 1 study with 1135 participants studied the effect of hyperbilirubinemia on GDM. The studies were conducted in various countries, including Korea (2 studies), China (5 studies), Kazakhstan (1 study), Japan (1 study), and the Netherlands (1 study).

**Table 1:** The characteristic of included cohort studies

Authors (Year)	Study design	Year(s) of study	Study population	Age ± SD (years)	Sample size	Bilirubin type	Type of measure DM/MS	Effect size (95% CI)	Outcome
S.-E. Lee et al., 2016	Retrospective longitudinal cohort	2006–2012	Korean adults	51.2 ± 8.3	22,084	PCB	DM a participant’s self-reported diagnosis on the questionnaire FPG ≥ 7 mmol/L HbA1c ≥ 6.5%.	HR for 1-SD increment: 1.26 (1.20–1.33) HRs by quartile: Q1 = (≤ − 28.6 (%)) = 1 Q2 = (− 28.5 – − 12.5 (%)) = 1.23 (1.00–1.51) Q3 = (− 12.4–11.1 (%)) = 1.69 (1.39–2.05) Q4 = (> 11.1(%)) = 2.13 (1.77–2.56)	T2DM
Xiao-Hong Li et al., 2017	Cohort	2011–2016	Healthy Chinese men	45.6 ± 12.7	1339	TBIL,DBIL,IBIL	AHA criteria	Model ORs TBIL Q1 (≤11.75 μmol/L) = 1 Q2 (11.76–14.30 μmol/L) = 1.32 (0.79–2.21) Q3 (14.31–18.12 μmol/L) = 0.87 (0.50–1.52) Q4 (> 18.12 μmol/L) = 0.61 (0.34–1.12) DBIL Q1 (≤2.09 μmol/L) = 1 Q2 (2.10_2.60 μmol/L) = 1.00 (0.61–1.63) Q3 (2.61_3.22 μmol/L) = 0.57 (0.32–1.02) Q4 (> 3.22 μmol/L) = 0.51 (0.28–0.92) IBIL Q1 (≤9.58 μmol/L) = 1 Q2 (9.59–11.76 μmol/L) = 1.16 (0.69–1.96) Q3 (11.77–14.90 μmol/L) = 0.92 (0.53–1.58) Q4 (> 14.90 μmol/L) = 0.58 (0.32–1.06)	MetS
Chaoqun Liu et al., 2016	Prospective Cohort	2013–2016	Chinese Han. Women receiving their first prenatal care prior to 16 weeks of gestation	28.31 ± 3.04	1135	TBIL,DBIL,IBIL	WHO criteria	Model RRs: TBIL T1 (1.7–4.8 μmol/L) = 1(reference) T2 (4.9–6.3 μmol/L) = 0.99 (0.82, 1.22) T3 (6.4–23 μmol/L) = 0.90 (0.82, 1.00) DBIL T1 (0.9–2.2 μmol/L) = 1 (reference) T2 (2.3–2.8 μmol/L) = 0.91 (0.75, 1.00) T3 (2.9–9.8 μmol/L) = 0.60 (0.35, 0.89) IBIL T1(0.1–2.6 μmol/L) = 1 (reference) T2(2.7–3.6 μmol/L) = 1.1 (0.76, 1.3) T3(3.7–13.9 μmol/L) = 0.86 (0.74, 1.0)	GDM
Sen Wang et al., 2017	Cohort	2011–2016	Healthy Chinese men and women	48.65 ± 11.05	32,768	TBIL	AHA criteria	ORs for Males Q1(≤ 9.90 μmol/L) = (reference) Q2(9.90–12.90 μmol/L) = 0.829 (0.758–0.908) Q3(12.90–16.80 μmol/L) = 0.814 (0.743–0.891) Q4(> 16.80 μmol/L) = 0.673 (0.613–0.739) ORs for Females Q1(≤ 8.00 μmol/L) = (reference) Q2(8.00–10.30 μmol/L) = 0.874 (0.762–1.001) Q3(10.30–13.40 μmol/L) = 0.761 (0.661–0.875) Q4(> 13.40 μmol/L) = 0.753 (0.653–0.867)	Mets
Y.J. Kwon et al., 2017	Prospective cohort	2001–2014	Korean adults 40 to 69 years of age	T2DM group: 54.3 ± 8.5 non-T2DM group: 51.6 ± 8.8	8650	TBIL	WHO criteria	ORs in men Q1 (< 0.47 mg/dL) = 1 (reference) Q1(< 8.03) Q2 (0.47–0.61 mg/dL) = 0.75 (0.55–1.03) Q2(8.03–10.43) μmol/L Q3 (0.61–0.82 mg/dL) = 0.73 (0.53–1.01) Q3(10.43–14.02) μmol/L Q4 (0.82–2.00 mg/dL) = 0.52 (0.36–0.74) Q4(14.02–34.2) μmol/L ORs in women Q1(< 0.36 mg/dL) = 1 (reference) Q1 < 6.15 μmol/L Q2 (0.36–0.46 mg/dL) = 0.73 (0.54–0.98) Q2(6.15–7.91) μmol/L Q3(0.46–0.62 mg/dL) = 0.54 (0.39–0.74) Q3(7.91–10.60) μmol/L Q4(0.62–2.00 mg/dL) = 0.65 (0.47–0.89) Q4(10.60–34.2) μmol/L	T2DM
Hao et al., 2020	Cohort	2009–2017	Kazakh permanent residents	40.33 ± 11.86	565	TBIL,DBIL,IBIL	JIS criteria	TBIL HR 0.35 (0.21–0.60) 0.36 (0.21–0.62) 0.38 (0.22–0.64) IBIL HR 0.31 (0.18–0.54) 0.39 (0.23–0.66) 0.31 (0.18–0.54)	Mets
Min Yang et al.,2019	Prospective cohort	2012–2014	all patients > 18 years of age and diagnosed for the first time with impaired glucose regular (IFG or IGT)	Q1: 60.4 ± 5.0 Q2: 61.6 ± 5.3 Q3: 62.7 ± 5.4 Q4: 64.0 ± 5.7	523	TBIL	WHO criteria	ORs Q1: Ref (< 8.2 μmol/L) =1 Q2: (8.3–11.1 μmol/L) = 0.83 (0.74–0.96) Q3(11.2–14.5 μmol/L) = 0.78 (0.68–0.90) Q4:> 14.6 μmol/L = 0.74 (0.64–0.87)	T2DM
Makoto Shiraishi et al., 2019	Retrospective cohort	2013–2018	middle-aged Japanese without Mets	44.8	8992	TBIL	JIS, AHA, WHO, IAS	HRs All: 0.70 (0.59–0.85) Men: 0.82 (0.66–1.01) Women: 0.60 (0.43–0.84)	Mets
Fan Zhang et al., 2020	Cohort	2014–2018	consecutive obese patients	29.85 ± 9.75 years	71	TBIL,DBIL,IBIL	hyperinsulinemia-euglycemic clamp technique (HEC) with glucose disposal rate (GDR, M value)	OR TBIL: 0.744 (0.590–0.938) DBIL: 0.575 (0.326–1.015) IBIL: 0.602 (0.413–0.878)	Insulin Sensitivity
Abbasi et al., 2015	Cohort	2015	Dutch population participate in the (PREVEND) study	49.4 ± 12.4	3381	TBIL	FPG level was ≥7.0 mmol/L (126 mg/dL), the random sample plasma glucose concentration was ≥11.1 mmol/L (200 mg/dL), they reported a physician diagnosis of T2DM, or they received insulin or oral hypoglycemic agents based on a central pharmacy registration.	OR TBIL:0.58 [0.39–0.84]; *P* = 0.005)	T2DM

*T2DM* Type 2 Diabetes mellitus, *Mets* Metabolic Syndrome, *GDM* Gestational diabetes mellitus, *FPG* fasting plasma glucose, *HbA1c* glycosylated hemoglobin, *AHA* American Heart Association, *WHO* World Health Organization, *JIS* Joint Interim Statement, *IAS* The International Atherosclerosis Society, *PCB* percentage change in serum bilirubin levels, *TBIL* Total bilirubin, *DBIL* Direct bilirubin, *IBIL* Indirect bilirubin

The studies included in the meta-analysis used different indicators to diagnose type 2 diabetes, including fasting plasma glucose (FPG) ≥ 7 mmol/L, HbA1c ≥ 6.5%, random sample plasma glucose concentration ≥ 11.1 mmol/L, WHO criteria, physicians’ diagnosis reports, and receiving insulin or other oral hypoglycemic drugs based on central pharmacy records. Various indicators were also used to diagnose MetS, including the American Heart Association, Joint Interim Statement (JIS) diagnostic criteria, joint interim statement of the International Diabetes Federation Task Force on Epidemiology and Prevention, the World Heart Federation, the International Atherosclerosis Society, and hyperinsulinemia-euglycemic clamp technique (HEC) with glucose disposal rate (GDR, M value). The World Health Organization criteria were utilized to diagnose GDM.

Different forms of bilirubin were investigated in the studies included in the meta-analysis, including PCB (percentage changes in serum bilirubin levels), total bilirubin (TBIL), direct bilirubin (DBIL), and indirect bilirubin (IBIL).

### Quantitative results

#### The effect of hyperbilirubinemia on the risk of type 2 diabetes

**Fig. 2:**
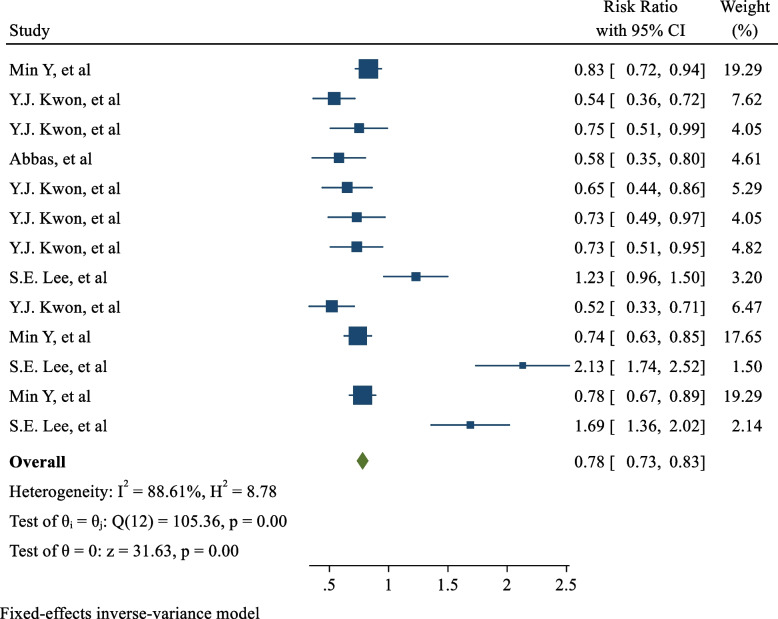
Forest and Funnel plot of the effect of bilirubin level on the risk of T2DM

The meta-analysis included 4 of the 10 studies that evaluated the effect of hyperbilirubinemia on the incidence of type 2 diabetes. The RR in these studies ranged from 0.52 (95% CI: 0.33, 0.71) to 2.13 (95% CI: 1.74, 2.52). Upon combining these studies, the pooled relative risk was 0.78 (RR: 0.78; 95% CI: 0.73, 0.83; I2: 88.61%; *P* heterogeneity < 0.001). These results suggest that the risk of developing type 2 diabetes in individuals with hyperbilirubinemia was approximately 22% lower than the risk in individuals without hyperbilirubinemia, as shown in Fig. 2.

#### The effect of hyperbilirubinemia on the risk of MetS

**Fig. 3:**
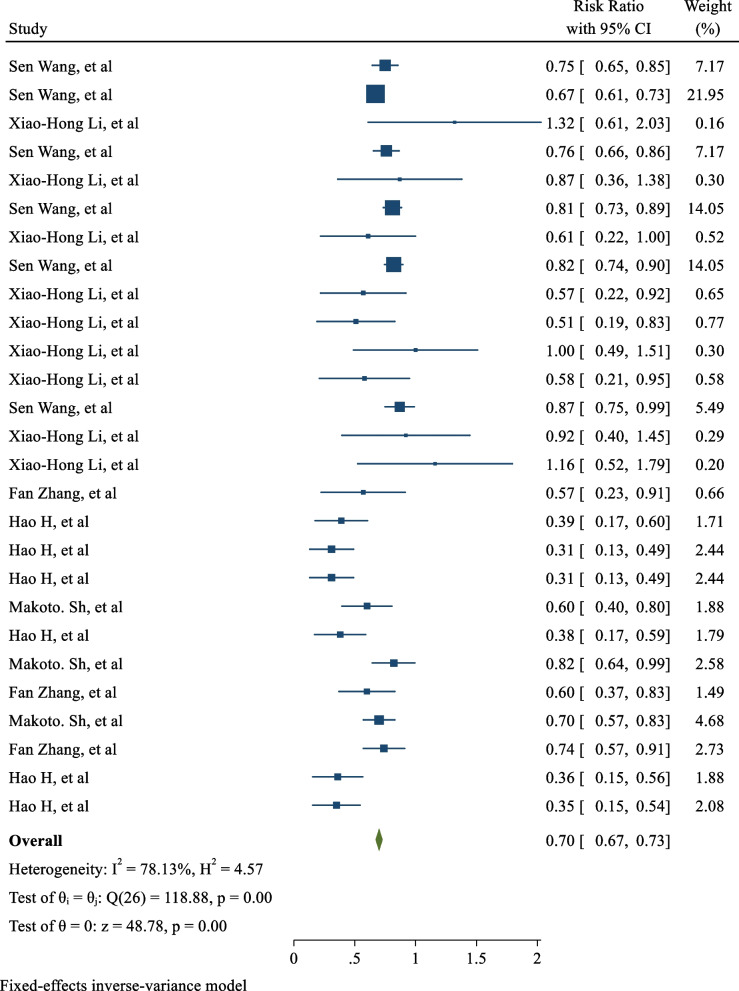
The Effect of hyperbilirubinemia on risk of MetS

Five out of the 10 included studies evaluated the effect of hyperbilirubinemia on the incidence of MetS. The relative risk measured in these studies ranged from 0.31 (95% CI: 0.13, 0.49) to 1.32 (95% CI: 0.61, 2.03). After combining these studies, the pooled relative risk was 0.70 (RR: 0.70; 95% CI: 0.67, 0.73; I2: 78.13%; *P* heterogeneity < 0.001). These findings suggest that the risk of developing MetS in individuals with hyperbilirubinemia was approximately 30% lower than the risk in individuals without hyperbilirubinemia, as illustrated in Fig. 3.

#### The effect of hyperbilirubinemia on the risk of GDM in women

**Fig. 4:**
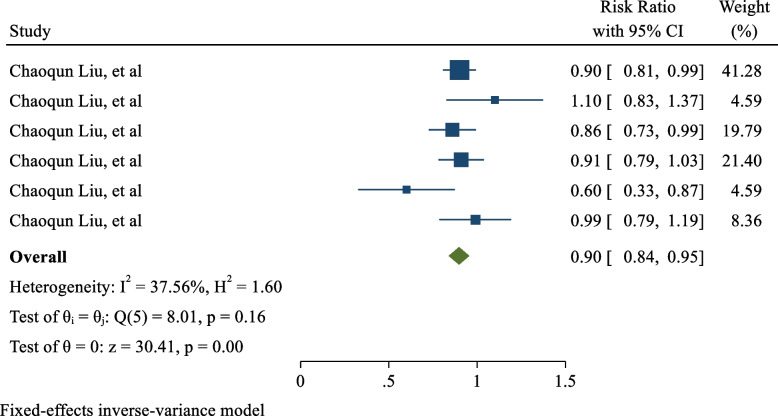
The Effect of hyperbilirubinemia on risk of GDM

In one of the included cohort studies, the association between bilirubin levels and the incidence of GDM was investigated. The study reported different effect sizes based on different indicators of bilirubin measurement and its various levels. Due to the limitations of the studies, these measurements were combined with one selected study. The results showed that the risk of GDM in women with high bilirubin levels was 0.90 (RR: 0.90; 95% CI: 0.84, 0.95; I2: 37.56%; P heterogeneity = 0.160), as shown in Fig. 4. These findings suggest that high bilirubin levels may be associated with a reduced risk of GDM, though further research is needed to confirm this relationship.

### Results of subgroup analyses

#### The effect of hyperbilirubinemia on the risk of type 2 diabetes based on different bilirubin levels and its measurement methods

The meta-analysis results revealed that different types and levels of measured bilirubin were associated with varying relative risks. Studies that evaluated TBIL reported a relative risk of 0.75 (RR: 0.75; 95% CI: 0.67, 0.77; I2: 41.58%; P heterogeneity = 0.089), whereas studies measuring percentage changes in bilirubin (PCB) reported a relative risk of 1.57 (RR: 1.57; 95% CI: 1.39, 1.76; I2: 33.90%; P heterogeneity = 0.077), as shown in Table 2.

**Table 2:** The association between of hyperbilirobimia and type 2 diabetes, gestational diabetes, and metabolic syndrome based on type of bilirubin measurements and bilirubin serum

Outcome	Variables	Categories	RR (% 95 CI)	Heterogeneity assessment				
				Between Studies			Between subgroup	
				I _square_	*P*_heterogeneity_	Q	Q	*P*_value_
T2DM	Type of Bilirubin Measurements	PCB	1.57 (1.39–1.76)	33.90%	0.077	15.49	38.30	0.0001
		TBIL	0.72 (0.67–0.77)	41.58%	0.089	10.09		
	Bilirubin Serum (Ref. ≤0.36 mg/dl)	> 0.36 mg/dl	0.73 (0.51–0.95)	–	–	–	7.93	0.030
		> 0.47 mg/dl	0.62 (0.52–0.71)	0.00%	0.420	3.87		
		NR	0.85 (0.79–0.91)	22.82%	0.088	8.09		
MetS	Type of Bilirubin Measurements	DBIL	0.40 (0.31–0.50)	37.06%	0.154	9.53	12.33	0.0001
		IBIL	0.67 (0.50–0.85)	19.77%	0.290	3.74		
		TBIL	0.73 (0.70–0.76)	56.30%	0.066	13.28		
	Bilirubin Serum	> 0.12 mg/dl (Ref. ≤0.12 mg/dl)	0.75 (0.72–0.79)	45.76%	0.049	15.82	22.47	0.0001
		> 0.029 mg/dl (Ref. ≤0.029 mg/dl)	0.62 (0.40–0.83)	24.95%	0.260	2.66		
		> 0.10 mg/dl (Ref. ≤0.10 mg/dl)	0.86 (0.75–0.97)	4.07%	0.370	3.13		
		NR	0.53 (0.48–0.59)	55.44%	0.052	14.80		
GDM	Type of Bilirubin Measurements	DBIL	0.86 (0.75–0.97)	26.40%	0.149	2.17	0.73	0.700
		IBIL	0.91 (0.79–1.02)	49.42%	0.129	2.46		
		TBIL	0.92 (0.83–1.00)	0.00%	0.420	0.65		
	Bilirubin Serum	> 0.12 mg/dl (Ref. ≤0.12 mg/dl)	0.92 (0.84–1.00)	0.00%	0.420	0.65	0.37	0.540
		> 0.029 mg/dl (Ref. ≤0.029 mg/dl)	0.88 (0.80–0.96)	17.15%	0.103	1.99		

*CI* confidence interval, *RR* Risk ratio, *NR* Not reported

Furthermore, the effect size varied based on the levels of bilirubin. If the level of bilirubin less than or equal to 0.36 mg/dL was considered as the reference level, the risk of developing type 2 diabetes in individuals with bilirubin levels greater than 0.36 or more than 0.47 mg/dL were 0.73 and 0.62, respectively (RR: 0.73; 95% CI: 0.51, 0.95; and RR: 0.62; 95% CI: 0.52, 0.71) (Table 2). These findings suggest that higher levels of bilirubin may be associated with a lower risk of developing type 2 diabetes.

#### The effect of hyperbilirubinemia on the risk of MetS based on different levels of bilirubin and its measurement methods

After combining the studies that evaluated the bilirubin level using DBIL, IBIL, and TBIL, the meta-analysis results showed that the association between bilirubin level and the incidence of MetS varied depending on the type of measured bilirubin. The relative risks were 0.40 (RR: 0.40; 95% CI: 0.31, 0.50; I2: 37.06%; P heterogeneity = 0.154) for DBIL, 0.67 (RR: 0.67; 95% CI: 0.50, 0.85; I2: 19.77%; P heterogeneity = 0.290) for IBIL, and 0.73 (RR: 0.73; 95% CI: 0.70, 0.76; I2: 56.30%; P heterogeneity = 0.066) for TBIL, as shown in Table 2.

Regarding different levels of bilirubin, the risk of developing MetS in individuals with bilirubin levels greater than 0.12 mg/dL was 0.75 compared to individuals whose bilirubin level was less than or equal to 0.12 mg/dL (RR: 0.75; 95% CI: 0.72, 0.79; I2: 45.76%; P heterogeneity = 0.049). The risk in individuals with bilirubin levels greater than 0.029 mg/dL was 0.62 compared to individuals whose bilirubin level was less than or equal to 0.029 mg/dL (RR: 0.62; 95% CI: 0.40, 0.83; I2: 24.95%; P heterogeneity = 0.260). The risk in individuals with bilirubin levels greater than 0.10 mg/dL was 0.86 compared to individuals whose bilirubin level was less than or equal to 0.10 mg/dL (RR: 0.86; 95% CI: 0.75, 0.97; I2: 4.07%; P heterogeneity = 0.370) (Table 2). These results suggest that higher levels of bilirubin may be associated with a lower risk of developing MetS.

#### The effect of hyperbilirubinemia on the risk of gestational diabetes based on different levels of bilirubin and its measurement methods

After combining the studies that evaluated the bilirubin level using DBIL, IBIL, and TBIL, the meta-analysis results showed that the association between bilirubin level and the incidence of gestational diabetes varied depending on the type of measured bilirubin. The RR were 0.86 (RR: 0.86; 95% CI: 0.75, 0.97; I2: 26.40%; P heterogeneity = 0.149) for DBIL, 0.91 (RR: 0.91; 95% CI: 0.79, 1.02; I2: 49.42%; P heterogeneity = 0.129) for IBIL, and 0.92 (RR: 0.92; 95% CI: 0.83, 1.00; I2: 0.00%; P heterogeneity = 0.420) for TBIL, as shown in Table 2.

Regarding different levels of bilirubin, the risk of developing gestational diabetes in women with bilirubin levels greater than 0.12 mg/dL was 0.92 compared to women whose bilirubin level was less than or equal to 0.12 mg/dL (RR: 0.92; 95% CI: 0.84, 1.00; I2: 0.00%; P heterogeneity = 0.420). The risk in women with bilirubin levels greater than 0.029 mg/dL was 0.88 compared to women whose bilirubin level was less than or equal to 0.029 mg/dL (RR: 0.88; 95% CI: 0.80, 0.96; I2: 17.15%; P heterogeneity = 0.103) (Table 2). These findings suggest that higher levels of bilirubin may be associated with a lower risk of developing gestational diabetes, but further research is needed to confirm this relationship.

### The results of publication bias

#### The effect of hyperbilirubinemia on the risk of type 2 diabetes

**Fig. 5:**
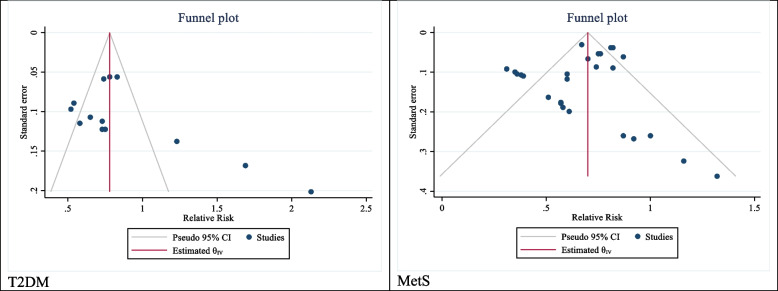
The funnel plot

Funnel plot and Egger test were used to check publication bias. The results of the test showed publication bias occurred in the results of investigating the association between the bilirubin level and the incidence of type 2 diabetes (B: 8.01; SE: 2.17; P: 0.0002). Non-parametric “Trim and Fill” analysis was used to investigate the effect of this bias on the overall results estimated in the present meta-analysis, the results of which showed it did not have a significant effect on the overall result. Using this analysis, it was estimated that if missing studies were considered, the overall estimate would be 0.89 with a confidence interval of 0.65 to 1.13 (Fig. 5).

#### The effect of hyperbilirubinemia on the risk of MetS

Funnel plot and Egger test were used to check publication bias. The results of the test showed publication bias did not occur in the results of examining the association between bilirubin levels and MetS (B: 0.59; SE: 0.622; P: 0.342). Non-parametric “Trim and Fill” analysis was used to investigate the effect of this bias on the overall estimated results in the present meta-analysis, the results of which showed if the missing studies were taken into account, the overall estimate would be equal to 0.62 with a confidence interval of 0.54 to 0.78 (Fig. 5).

## Discussion

The findings of this systematic review and meta-analysis suggest that higher levels of bilirubin may be associated with a lower risk of developing type 2 diabetes, MetS, and gestational diabetes. These results may have important clinical implications in terms of identifying potential therapeutic targets for these conditions. The study by Ndisang et al. that you mentioned also supports the role of bilirubin as an antioxidant and its potential role in reducing oxidative stress. Further research is needed to understand the underlying mechanisms behind these associations and to determine whether interventions that increase bilirubin levels can be used as a potential treatment for these conditions [1]. The findings from animal studies showing improved insulin synthesis and sensitivity, increased expression of GLUT4, and protection of pancreatic B cells against oxidative stress and damage further support the potential role of bilirubin in reducing the risk of type 2 diabetes. These findings suggest that bilirubin may have a beneficial effect on glucose metabolism and insulin sensitivity, which are key factors in the development of diabetes. However, it should be noted that the findings from animal studies may not necessarily translate to humans, and further research is needed to confirm the potential therapeutic effects of bilirubin in humans [2, 23]. Bilirubin has been shown to have anti-inflammatory and antioxidant properties, which may help reduce systemic oxidative stress and inflammation, both of which are known to contribute to insulin resistance. By reducing oxidative stress and inflammation, bilirubin may help improve insulin sensitivity and reduce the risk of developing insulin resistance and type 2 diabetes. In addition, bilirubin has been shown to have a protective effect on endothelial cells, which play a key role in regulating blood glucose levels and insulin sensitivity. Overall, these findings suggest that bilirubin may have multiple beneficial effects on glucose metabolism and insulin sensitivity, and further research is needed to better understand the mechanisms behind these effects [10, 24]. The study by Abbasi et al. [11] using the Mendelian randomization approach provided additional evidence for the potential protective effect of bilirubin on the development of type 2 diabetes. The study found that a common genetic variant (rs6742078) of the UGT1A1 gene, which is involved in bilirubin metabolism, was strongly associated with an increase in the circulating level of total bilirubin and a decrease in the risk of type 2 diabetes. This finding suggests that the increase in bilirubin levels may be causally related to the reduction in the risk of developing type 2 diabetes. In addition, the study found an inverse association between plasma bilirubin levels and type 2 diabetes, further supporting the potential protective effect of bilirubin on the development of this condition.

The observation that diabetic patients with Gilbert’s syndrome, a genetic disorder that causes elevated levels of bilirubin, have reduced oxidative stress markers also supports the potential role of bilirubin in reducing oxidative stress and inflammation, both of which are key factors in the development of type 2 diabetes. However, it is important to note that the findings from observational studies should be interpreted with caution, as they may be subject to confounding factors and reverse causation [11, 24].

In addition, the meta-analysis showed that increasing bilirubin levels reduced the risk of MetS. This finding is consistent with previous evidence suggesting that serum bilirubin has anti-inflammatory and antioxidant functions, which can improve vascular endothelial function and increase insulin sensitivity in tissues. These effects may help reduce the risk of developing MetS by improving glucose metabolism, reducing inflammation, and reducing oxidative stress. Overall, these findings support the potential role of bilirubin as a protective factor against the development of type 2 diabetes and MetS [14, 25]. Bilirubin can have antioxidant effects by suppressing the oxidation of lipids and lipoproteins [26]. It also has anti-atherogenic effects and plays a role in pathways related to the vascular structure and reactivity [27]. The high heterogeneity observed in the combined studies is a common issue in meta-analyses and can be due to various factors, such as differences in study populations, study designs, and measurement methods. In this systematic review, subgroup analysis was performed based on the type of bilirubin and its different levels, which helped to identify the main sources of heterogeneity and to better understand the association between hyperbilirubinemia and type 2 diabetes. The subgroup analysis showed that increasing PCB in contrast to TBIL increased the risk of developing type 2 diabetes in the future. Moreover, the results indicated that increasing the serum bilirubin level to more than 0.47 mg/dl compared to the increase to more than 0.36 mg/dl decreased the risk of type 2 diabetes. According to the results of the subgroup analysis, increasing direct bilirubin compared to indirect one leads to a greater reduction in the risk of MetS. Also, each of the direct and indirect bilirubin variables has a more protective effect on the risk of MetS than total bilirubin. If the amount of bilirubin is greater than 0.12 mg/dl, the risk of MetS will be 0.75 for people whose bilirubin is less than or equal to 0.12 mg/dl [28, 29].

In other words, the risk of disease in people with bilirubin levels more than 0.12 mg/dl is 15% lower. In people with the bilirubin level more than 0.029 mg/dl, the risk of MetS is 0.62 compared to people whose bilirubin is less than or equal to 0.029 mg/dl. The risk of MetS in values higher than 0.10 mg/dl is 0.86 compared to values less than or equal to 0.10 mg/dl. According to the findings of two studies, with the increase in the serum bilirubin level, the risk of MetS decreased in women more than men [30, 31]. The results of the study of Y.J. Kwon et al. [32] indicated the risk of T2DM was different between men and women based on different amounts of total bilirubin. In the bilirubin range of 0.46–0.62 mg/dL, the risk of developing T2DM is lower in women compared to men, and in the bilirubin range of 0.62–2.00 mg/dL, the risk of developing T2DM was lower in men. More studies are necessary about the effect of increased bilirubin on the risk of T2DM and MetS according to gender.

The results of a prospective cohort study by Chaoqun Liu et al. showed high serum direct bilirubin levels in the second trimester of pregnancy reduced the risk of gestational diabetes [23]. Bilirubin was proven to have a protective effect against factors affecting cardiovascular diseases such as high blood pressure, MetS and obesity through its antioxidant potential [33, 34]. Although the exact pathogenesis of diabetes is not fully defined, in vitro and in vivo studies and clinical evidence indicate the important role of the heme catabolic pathway in the development of this disease [35, 36]. Some studies reported the levels of oxidative stress biomarkers including DNA damage biomarkers and lipid peroxidation products increased in women with GDM [37]. Increasing the antioxidant capacity of serum, such as increasing the amount of bilirubin, is thought to help treat diabetes and its complications [38].

One of the limitations of this study was lack of the sufficient number of articles related to the association between bilirubin levels, MetS and T2DM in different genders. On the other hand, the number of studies investigating the association between bilirubin levels and the incidence of gestational diabetes in women was limited. Another limitation of this study was the inability to conduct subgroup analyses based on different definitions of MetS due to the limited number of studies in this regard. The fact that the data extracted and the studies conducted were limited to Asian countries is also a limitation of this study. This is because the majority of studies in this area worldwide have been conducted in these countries. Regarding the generalizability of the results of this meta-analysis, it can be said that they are generally applicable. However, it is possible that the generalizability is more relevant to the lifestyle and health behavior of the specific regions where the studies were conducted.

## Conclusion

Based on the results of this meta-analysis, it can be concluded that high levels of bilirubin may have a significant protective effect against type 2 diabetes and MetS. However, more studies are needed to confirm these findings and to determine the underlying mechanisms behind these associations. Regarding gestational diabetes, the meta-analysis did not find a significant association between bilirubin levels and the risk of developing this condition. However, it is important to note that the number of studies included in the analysis of gestational diabetes was relatively small, and more studies with a larger sample size are needed to investigate the potential association between bilirubin levels and the risk of gestational diabetes. Overall, this meta-analysis provides evidence for the potential protective effects of bilirubin against the development of type 2 diabetes and MetS, but further research is needed to confirm these findings and to determine the clinical implications of these associations.

## Data Availability

Not applicable.

## Abbreviations

MetS: Metabolic syndrome
T2DM: Type 2 Diabetes mellitus
GDM: Gestational diabetes mellitus
WHO: World Health Organization
BMI: Body mass index
NCEP: National Cholesterol Education Program
CAD: Coronary artery disease
IDF: International Diabetes Federation
NOS: Newcastle-Ottawa Scale
HEC: Hyperinsulinemia-euglycemic clamp
GDR: glucose disposal rate
FPG: Fasting plasma glucose
